# An Investigation of the Free Energy Principle for Emotion Recognition

**DOI:** 10.3389/fncom.2020.00030

**Published:** 2020-04-22

**Authors:** Daphne Demekas, Thomas Parr, Karl J. Friston

**Affiliations:** ^1^Department of Mathematics, University College London, London, United Kingdom; ^2^Wellcome Trust Centre for Neuroimaging, University College London, London, United Kingdom

**Keywords:** free energy (Helmholtz energy), artificial intelligence, emotion recognition (ER), active inference, Markov blanket (MB), bayesian brain

## Abstract

This paper offers a prospectus of what might be achievable in the development of emotional recognition devices. It provides a conceptual overview of the free energy principle; including Markov blankets, active inference, and—in particular—a discussion of selfhood and theory of mind, followed by a brief explanation of how these concepts can explain both neural and cultural models of emotional inference. The underlying hypothesis is that emotion recognition and inference devices will evolve from state-of-the-art deep learning models into active inference schemes that go beyond marketing applications and become adjunct to psychiatric practice. Specifically, this paper proposes that a second wave of emotion recognition devices will be equipped with an emotional lexicon (or the ability to epistemically search for one), allowing the device to resolve uncertainty about emotional states by actively eliciting responses from the user and learning from these responses. Following this, a third wave of emotional devices will converge upon the user's generative model, resulting in the machine and human engaging in a reciprocal, prosocial emotional interaction, i.e., sharing a generative model of emotional states.

## Introduction

How does the mind formulate ideas about the world around it? How does the spontaneous interpretation of human emotion guide our behavior and beliefs, and how are these emotional states predicted and understood? Recent advances in theoretical neurobiology are shedding light on emotionally intelligent artifacts—advances that may provide insight into the future of psychiatry and human-machine interaction. In this paper, we focus upon developments afforded by the free energy principle.

The free energy principle derives from a view of the brain as a statistical machine. This concept originated from the German physicist Hermann von Helmholtz, who in 1866 suggested that the brain performs unconscious inference (von Helmholtz, 1866). The implicit Bayesian brain hypothesis addresses the divergence between the inferences drawn by the brain, and the “hidden” surroundings in the external environment. These are hidden in the sense that they must be inferred vicariously through sensory samples. The brain is Bayesian, so to speak, because its prior knowledge and beliefs act as a starting point from which it can perform statistical inference on the hidden states of the environment, that underwrite perception and action (Friston, 2012).

In contemporary research, a Helmholtz machine is a statistical engine that can infer the *probable causes* of sensory input. These machines can learn to perform inferences on their own, with a bottom-up recognition model that infers causes from sensory input, and a top-down generative model which trains the recognition model (Dayan et al., 1995). Building upon this, the free energy principle was introduced to describe how living, self-organizing agents have evolved to exist in a confined “state space” with a bound on their long-term entropy (Friston, 2012). The value of the free energy principle is its mathematical rigor, which allows for the modeling of many different forms of behavior across species. For the purpose of this review, we focus on an application of this principle to the inference of human emotion, where we define emotion as simultaneously a result of sensation and a cause of action. As a result, we can begin to see how emotions guide human perception, beliefs and behavior and how the communication and inference of emotions may be simulated with intelligent devices.

We hypothesize that the foundations of the free energy principle and active inference can give rise to more advanced emotion inference devices, and we unpack the underlying principles and advantages of these potential developments.

## Theoretical Overview

The free energy principle conjectures the imperative that underwrites adaptive behavior in biological systems—from split second decisions through to evolution and generations. The principle describes how biological agents restrict themselves to a limited number of (likely) sensory encounters by continuously updating expectations about their environment and acting to minimize sensory entropy and maintain order (Schwartenbeck et al., 2015; Friston, 2009). The key idea is that their brains encode a Bayesian recognition density, with neural dynamics determined by the internal (generative) model that predicts sensory data based upon alternative hypotheses about their causes. These dynamics are interpretable as an inference of the probable cause of observed sensory data that “invert” the generative model, finding the best “fit” to the environment. Crucially, there are two ways of ensuring the model and environment are a good fit to one another. The first is the optimization of the recognition density to capture the most probable environmental configurations. The second is by changing the environment through action to make it more consistent with model predictions. Via these two (active inferential) mechanisms, the brain will self-organize to avoid improbable (under the generative model) sensory encounters; i.e., those associated with existential risk.

The objective function that is optimized through Bayesian inference is the surprisal (negative log probability) of sensory data under a model. This quantifies the fit of the model to the data (Schwartenbeck et al., 2015). The above suggests that action and perception are both engaged in minimizing surprisal. Interestingly, the average surprisal is the entropy alluded to above. Minimizing the former on average corresponds to the minimization of sensory entropy and ensures the occupancy of a small number of highly probable states—i.e., self-organization. While surprisal is a difficult quantity to directly assess, it is bounded by a more tractable quantity: variational free energy. As such, minimizing free energy ensures an upper bound on surprisal and entropy.

Computationally, free energy minimization occurs in relation to a generative model through performing active inference. This allows for a continuous and reciprocal optimization of sensory information (via action) and expectations (via inference). Our focus here is on how these fairly abstract notions translate to the computations that underwrite a specific problem—that of social interaction and inference about emotional states. When it comes to social interactions, active inference allows for the communication of emotions through action (through generating speech or facial expressions) and inference about another's emotional state from sensory input such as auditory signals or visual impressions of facial expressions (Vogt et al., 2008). The active inference approach assumes a person employs a predictive model of what they would expect from a social interaction in terms of emotional content, what alternative emotional content predicts in the auditory or visual domain, as well as what would cause these expectations to change.

An active inference approach to emotion recognition, other than being useful for industrial applications, is valuable for psychiatric research and practice in order to understand, model, and potentially help recovery from psychological false inferences that lead to altered perception of emotions and behavior.

### Markov Blankets

The free energy principle is underwritten by the conditional dependencies implied by a Markov blanket, which is a mathematical structure that distinguishes self-organizing systems from the external world. Markov blankets formalize the recurrent interactions between internal, sensory, active, and external states and explain how it is possible for inference to occur in multiple (and nested) spatial and temporal scales. In brief, a Markov blanket is a statistical boundary that renders everything outside the blanket conditionally independent of everything inside of it (Pearl, 2014). A common rhetoric used to unpack this is that the blanket states of a given internal state are the parents (things that cause it), children (things that it causes), and parents of its children. The parents of internal states are the sensory states that mediate the influence of the outside world, and their children are the active states that mediate their influence on the outside world. The Markov blanket framework is essential in understanding how any agent is able to achieve autonomy and actively interact with its environment, including other agents.

**Figure 1:**
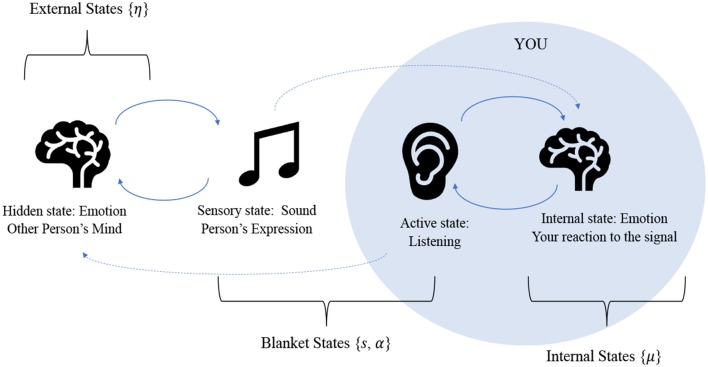
This figure represents the relationships between the various states due to the Markov blanket partition: active states are directly influenced by internal states and sensory states are directly influenced by external states. Only through these influences are the internal and external states are coupled to one another. The figure illustrates the process of an emotion being communicated between two human agents. Another person (external state) expresses an emotion audibly, which furnishes sensation for the agent. The agent performs inference, by listening and interpreting the sound based on prior knowledge, experience, and contextual expectation, performing motor action if needed (such as listening attentively). This leads to the agent forming an understanding and updating their internal states accordingly. Here, our classification of emotional states is quite simple: emotions are either part of an agents' “internal states” or “external states” (which are just the internal states of another agent) meaning they are a feature of the self or the other. In this sense, emotional states exist as the result of a sensation (i.e., as an inference about the cause of that sensation). In terms of communication, this then means that the awareness of other people's emotional states results in a kind of advantage for a human, in the form of a more accurate generative model for the reasons behind other humans' actions. Clearly this is a very coarse-grained view of emotional inference. For greater detail on the intricacies of emotion research, please see (LeDoux, 2000; Seth, 2013; Smith and Lane, 2016; Panksepp et al., 2017).

All living systems have a Markov blanket, because in order for a system to be alive, it must be endowed with some degree of conditional independence from its environment, or else it would be indistinguishable from it. What results from this partition is 4 sets of states: 2 blanket states, which are sensory and active states, that insulate internal states from external states (Ramstead et al., 2018). Figure 1 illustrates the structure of a Markov blanket as formulated for the emotional inference setting we have in mind. The key thing to draw from this is that internal states appear to infer external states vicariously, as they are only influenced by external states via blanket states.

### Self-Organization

Systemic states are variables that interact with each other, at a given time and within a given environment. If the system is disordered, the interactions between states lack any recognizable pattern, symmetry, or rules. Self-organization is the process by which the local interactions between systemic states spontaneously develop order, usually due to negative feedback mechanisms; where deviations from an ordered configuration are corrected, resisting the dispersive influence of random fluctuations (Nicolis and Prigogine, 1977; Kauffman, 1993; Kelso, 1995; Pasquale et al., 2008; Bruineberg and Rietveld, 2014; Halatek et al., 2018; Friston, 2019). The theory goes that brains are systems with the ability to self-organize to attracting states (i.e., goals) or equivalently non-equilibrium steady states.

Imagine that every single *state of being* has a position in an abstract state space. There are 4 important kinds of states (i.e., dimensions) in this space: sensory states (e.g., the sound of a voice), active states (e.g., listening^1^), internal states (e.g., thoughts, feelings), and environmental states (e.g., location, context). These states are by definition the partitions afforded by Markov blanket.

Now imagine different biological agents, like dogs, cats, humans, and bacteria. These different agents have different phenotypic traits, different abilities and different goals, and because of this, each will occupy a different limited repertoire of states in state space (Friston, 2019). The classical example is a fish in water, which is an (environmental) state of a fish that is necessary for the fish's traits to be sustained. Once outside of the water, the fish can no longer possess the active trait of swimming, or the internal state of oxygenation, and so it ceases to be a living fish (Friston, 2019). This example highlights the importance of surprisal minimization, as being in water is a less surprising state in which to find a fish compared to finding it out of water.

From this, we can characterize the interactions that this agent has with the world around it, i.e., the exchange between environmental and internal states, which are mediated by sensory input (sensory states) and action (active states). These interactions will be self-organizing in a way that ensures that the pattern of interactions between these states is preserved over time. In other words, a living, self-organizing agent is spontaneously attracted to a certain set of states and will tend to converge to those states with a *low entropy* (e.g., the fish will tend to be found in water). This convergence appears mathematically analogous to the minimization of free energy, an upper bound on uncertainty, or surprisal. In terms of emotional states, free energy minimization allows for a generalized emotional homeostasis, or emotional balance.

### The Free Energy Principle

We define a state as a set of possible events (i.e., each event is a possible realization of the state). When a (living) agent occupies some state, each event in the state is assigned a probability, with the probability over all events summing to one. This distribution of probabilities is associated with different levels of certainty, or equivalently precision (inverse variance) negentropy. These quantify how peaked the distribution is.

If very precise, we can be confident that a specific event will occur. If imprecise (i.e., flat), probability mass is shared over a greater number of plausible events. In this case, an agent will be uncertain about whether or not an event will occur, meaning it will not know precisely what to expect, and this uncertainty is equivalent to a large number of *potential* occurrences.

The entropy of a distribution is the average surprisal of all states or events. The free energy principle places an upper bound on surprisal, therefore bounding the entropy and ensuring a degree of certainty about what is going to happen (Schwartenbeck et al., 2015; Friston, 2009).

With a large free energy, there is room for strange (improbable) things to happen to the agent that might threaten its existential integrity. It has a wide range of sensory states that it can occupy and a lot of uncertainty about the environment. This goes against the idea above of a structure that guarantees that phenotypic bounds are not transgressed. The free energy principle (Box 1) requires that any adaptive change will minimize the agent's free energy. In other words, living agents will continuously restrict their available sensory states by placing an upper bound on how surprising sensory states can be, on average.

Box 1 **Free energy minimization.**

See Friston, Karl *Trends in Cognitive Sciences*, vol. 13, no. 7, 2009 for a full mathematical description of the free energy principle in the context of continuous state-space generative modes. In short, states are mathematically described in terms of random variables that have defined flows with deterministic and random contributions. These flows, and the way in which these variables generate observable data, make up the generative model. Holding beliefs of this sort—that describe the generation of sensory data—affords the opportunity to invert this process to draw inferences about the environmental causes.

Minimizing free energy is equivalent to minimizing a Kullback-Leibler (KL) divergence between two probability densities that are specified by their sufficient statistics, μ which are supposedly encoded in the brain. The KL divergence is between a conditional density for the causes of sensations, *p*(η|*s*) and a recognition density, *q*(η|μ). The free energy principle states that action α and sufficient statistics (i.e., internal states) μ change to minimize free energy, resulting in the recognition density that approximates the conditional density.

Free energy is defined as the sum of the KL divergence and surprisal, where surprisal is now a function of the agent's sensory input (*s|m* where *m* is the agent). Thus, free energy minimization makes the KL divergence small (but always positive), which results in the free energy being a tight upper bound on surprisal, by definition:

$$\begin{array}{c} \text{FreeEnergy}=\text{KLDivergence}+\text{Surprisal}\\ F=D(q(\eta |\mu )||p(\eta |s,m)-ln(p(s|m)) \end{array}$$

A state *x* ∈ {*x*_1_…*x*_*n*_} Probability distribution: *p*(*x*) = *Cat*(**x**)

Surprisal: ℑ(*x*) = −*l*n(*p*(*x*)) Entropy: $H\left(X\right)=E\left[ℑ\left(x\right)\right]=\underset{i}{\sum }p\left(x_{i}\right)ℑ\left(x_{i}\right)$

**Figure 2:**
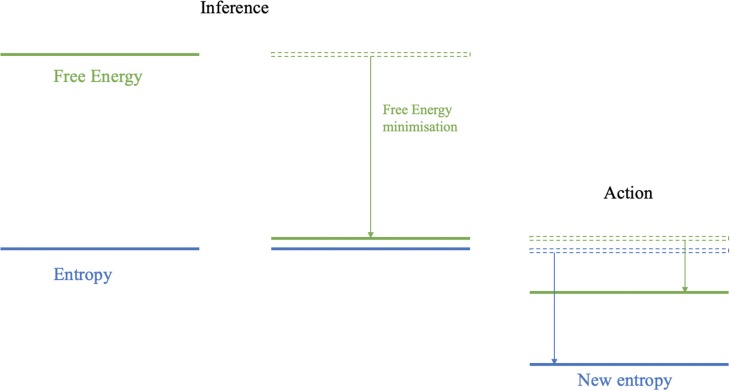
Entropy is the average surprisal and represents the uncertainty over states at a given moment in time. The free energy is a quantity that will always bound the surprisal from above. In the inference step (i.e., perception), free energy is minimized, i.e., the upper bound on surprisal is “pushed” down by changing beliefs or predictions concerning sensory outcomes. This minimization enforces a tight upper bound on the entropy. In the active (i.e., action) step sensations are sampled from the external states that reduce surprisal and thus ensure an upper bound on entropy.

In Figure 2, the states and their probability distribution are modeled continuously, and this conceptualization raises an important point when it comes to applying this model to emotional inference. In our discussion so far, we have treated the problem of inferring emotions to be a categorical problem. To some extent, this is licensed by the way in which we describe emotions; ascribing labels such as “happy” and “sad.” However, we also know that our experience of emotions allows for mixtures of these categories to co-exist. This implies emotions are most likely more complicated than states in a discrete set, and the difference between a discrete and continuous distribution of possible emotional states (i.e., a discrete probability mass function over a set {happy, sad, tired} or a continuous probability density function over a spectrum of these states) defines the difference between these two conceptions of emotional “states.” Thus, the prior beliefs and inferences of the emotional device can be imagined as more of a “position” in emotional space, rather than an exact state. Despite the fact that we will treat emotions as discrete states throughout this paper for the sake of simplicity, it is important to remember that a continuous description is possible and may co-exist. It is also likely that, if represented discretely, emotional states live on a discretized version of a continuous spectrum. Furthermore, different axes of emotional states may be factorized from one another [c.f., mean-field assumptions (Friston and Buzsáki, 2016)]. This facilitates feeling happy and disgusted at the same time.

### Generative Models and Active Inference

Given that the environment is only seen by agents through a Markov blanket, an agent's best bet at survival is to generate probabilistic inferences about its environment via sensory signals. In predictive coding formulations of free energy minimization, this may proceed through generating predicted observations (i.e., predicted sensory states) from an internal generative model and comparing these to observed states. The results of this comparison comprise a prediction error and can be thought of as a simple form of free energy. These prediction errors can either be used to update beliefs generating predictions (i.e., perception) or they can be used to sample the environment in a way that conforms to predictions (i.e., action) (Feldman, 2009). For an accurate prediction of what the environment entails, the agent must minimize free energy or prediction errors (Conant and Ashby, 1991; Ashby, 1947; Conant and Ashby, 1970; Mansell, 2011; Solway and Botvinick, 2012). Mathematically, this is the same as maximizing model evidence. In the context of machine learning, this is equivalent to maximization of an evidence lower bound or “ELBO” (Winn and Bishop, 2005).

An *intelligent adaptive agent* will continuously evince this inference process to create new assumptions and update prior beliefs about the world (Friston et al., 2017a; Parr and Friston, 2017). It will first sample the environment based on sensory input, then act on the environment in a purposeful way, and then update its internal model. By acting on the environment (increasing range of view, jumping to see higher, squinting to see better; all examples of acting in response to visual input), it will effectively change sensory input (seeing more, seeing better), which directly follows from the structure of the Markov blanket. This exchange is extremely important to the continuous minimization of free energy, because it becomes an autonomous cycle, recapitulating the “action-perception” cycles that underwrite modern cognitive science (Fuster, 1990).

Agents that obey the free energy principle via “adaptive action” are said to engage in active inference. This means the agent will perform the actions associated with the least free energy, i.e., the actions that confirm its causal predictions of the most likely sensory states. In other words, the minimization of free energy requires the agent to act in order to avoid surprising sensory encounters, thereby increasing the accuracy of its sensory data (Friston, 2009). An introduction to the mathematics of active inference can be found in Box 2.

Box 2 **Active inference.**

See Solopchuk, Oleg, “*Tutorial on Active Inference”* for an in-depth mathematical description of active inference. In brief, one needs to make an approximation to the posterior purely based on sensory states and free energy minimization and then use this approximation to infer the conditional probability of hidden states. Generally, this approximation is done by introducing an arbitrary distribution, say *q(*η*|*μ*)*, which depends on the internal states, and is integrated within the generative model so that free energy becomes a functional of *q(*η*|*μ*)*. Then, by minimizing free energy, we bring our approximate distribution closer to the posterior. When free energy = surprisal, then our arbitrary distribution is exactly this posterior (Solopchuk, 2019).

Note that this does not happen only once but over and over again at each time step, and also that within this interval of time steps, the agent is free to act on its environment, thus changing the sensory input over the time steps that follow. However, actions are not independent, in fact they follow each other in some kind of a plan, called a policy, which is a series of actions oriented toward a goal which is distant in time (Solopchuk, 2019). Therefore, the brain will plan a policy and integrate this in its generative model and its process of inference. There will be many possible actions and many future time points, and therefore there will be several potential policies. The generative model will consider every plausible policy and pick a policy that has the smallest expected free energy in the future (Solopchuk, 2019).

A final intricacy to note is that since the model doesn't have access to future observations, it must therefore guess what they could look like based on a given policy (Solopchuk, 2019).

In summary, this section has outlined the principles that underwrite active inference. Crucially, this rests upon a separation between the internal and external components of a generative model, such that the external world (e.g., other people) must be inferred vicariously. Given that the problem raised in the introduction—“how can we draw inferences about the emotional states of others?”—relies upon the notion of self and not-self (other) and upon the idea of vicarious inference, this implies the formalism afforded by active inference is well-suited to address this problem. In the next section, we provide an overview of some of the applications of active inference, with a special focus on inference about another's emotional state.

## Emotional Inference, Selfhood, and the Human Brain

Up until now, our formulation of the free energy principle—and corresponding concepts—have been articulated in terms of any self-organizing biological agent. Now we turn to the human brain and consider how active inference provides a computational architecture that allows the brain to distinguish between the self and other—and make inferences about emotional states in particular.

According to theory of mind, the prerequisite for any inference about another is the ability to form a clear distinction between the “self” and “other” (Zahavi, 2008) This distinction can be modeled perfectly with a Markov blanket, in which the “self” consists of internal states, and the external states consist of everything other than the self (Seth and Friston, 2016). However, this does not mean that the internal states “know” that they are internal. To associate active inference with a minimal sense of self, requires a bit more work (Zahavi, 2008; Limanowski and Blankenburg, 2013; Seth, 2013;Williford et al., 2018).

From the point of view of active inference, things only exist as a label for a hypothesis or inference about hidden states (i.e., hidden behind the Markov blanket). This means that, in order for the “self” to exist, there must be a generative model that has “self” as a hypothesis. Not all living agents have this model; viruses, for example, may not have self as a hypothesis. When we have “experiences,” we hypothesize that it is the self that is “experiencing,” as oppose to the other.

When it comes to emotions, if an emotion provides a simple explanation for the myriad of social cues in the environment, then the hypothesis of a given emotional state will equip the brain with a better generative model; namely, a model that best reflects the fact that much of the sensory evidence at hand is generated by an agent with emotional states (Seth and Friston, 2016).

For the ability to disambiguate an emotional states, it is necessary for an artifact to have a good generative model for emotional inference, with the capacity for emotional perspective. It is generally considered that emotional inference entails some predictions about interoceptive states, that may or may not be realized through autonomic reflexes and, literally, provide the basis for “gut feelings” (Ainley et al., 2012, 2016; Barrett and Simmons, 2015; Palmer et al., 2015; Fotopoulou and Tsakiris, 2017).

When it comes to the interaction between two self-organizing agents that both possess their own Markov blankets, the external states from the perspective of one agent are the internal states of the other. The existence of this shared narrative presents the opportunity of building emotionally responsive artifacts that can be both observers and recipients of emotional observation and interaction (Friston and Frith, 2015a).

Moreover, it is important to consider all of the ways that the brain and its generative model can go awry, so that we can pre-empt this in developing artificial systems. Interestingly, conditions seen in psychiatry are almost exclusively about the self in relation to others (Seth and Friston, 2016). For example, anorexia nervosa is a disorder of how others perceive the self physically, schizophrenic symptoms are often related to feeling threatened (or controlled) by others, and agoraphobia is the unwillingness to go outside for fear of the presence of other people. In general, psychiatry is concerned with the failure of interpersonal inference, and this necessarily has an emotional aspect to it. Under the active inference framework, these psychiatric symptoms occur due to a chemical imbalance which results in the failure of emotional inference. This underwrites the importance of a hierarchical generative model capable of optimizing beliefs about precision in developing synthetic systems capable of determining emotional states of others (Limanowski and Blankenburg, 2013; Corlett and Fletcher, 2014; Friston, 2017; Powers et al., 2017; Limanowski and Friston, 2018; Rae et al., 2019).

### A Model of Neural Structure

Evolutionary Systems Theory (EST)—under the Hierarchical Mechanistic Mind (HMM) model—considers how mechanisms such as evolution, enculturation, development, embodiment, and behavior act on different time scales to shape a reciprocal brain-body-environment system and engender the structure and function of the brain (Badcock et al., 2019).

The human brain is a self-organizing hierarchy of neurons that interact bidirectionally over multiple spatiotemporal scales. The lowest levels of the hierarchy are those on the periphery of the brain, interfacing with the peripheral nervous system, which in turn interfaces with muscles and sensory receptors. Higher cortical levels are further removed from primary cortical areas in the brain. Some of these higher regions, notably the hippocampus, are associated with greater plasticity than some others. This ensures they respond flexibly to input received from lower levels (Badcock et al., 2019). Through a complex mixture of short and long range connections on different scales, these cortical levels interact and, from these interactions, human cognition, and behavior emerge (Box 3).

Box 3 **Pyramidal cells.**

For a more detailed explanation see Friston et al., 2016b “*The Dysconnection Hypothesis”*.

Neurobiologically, the passing of messages between hierarchical cortical areas is mediated by pyramidal cells, among the largest neurons in the brain. Pyramidal cells exist both in superficial regions of the neocortex as well as deeper layers, and cells in different regions play different roles in the process of cognition and sensory processing. Under predictive coding models of neuronal message passing conditional expectations are encoded by deep pyramidal cells at each level of the cortical hierarchy that convey predictions downward to suppress errors, which are encoded by the superficial pyramidal cells (Bastos et al., 2012; Shipp, 2016; Friston et al., 2017b). This organization explains why superficial pyramidal cells have so many synaptic gain control mechanisms (NMDA receptors, D1 dopamine receptors, etc.), in order to set the gain or precision of prediction errors—thought to be encoded by superficial pyramidal cells (Pinotsis et al., 2014; Kanai et al., 2015; Shipp, 2016).

The HMM and EST view the brain as a fractal and nested modular hierarchy, which is self-similar across scales and can be modeled mathematically by a system of nested Markov blankets. This requires that the interaction between these nested hierarchical levels must itself be optimized. In the brain, this organization may be a key role of neuromodulatory systems, which act to control the gain of signals passed up or down the cortical (and subcortical) hierarchy.

From the perspective of a generative model, the confidence with which a variable at a lower hierarchical level may be predicted by that at a higher level is quantified by the precision of the distribution mapping the latter to the former (Clark, 2013b). In predictive coding, this reduces to the precision of prediction errors that are sent from one level to the next—thought to be encoded by superficial pyramidal cells (Feldman and Friston, 2010). The brain's neuromodulatory mechanisms appear to perform gain control operations in a manner analogous to the precision optimization (see Box 4). This underwrites attentional processing in computational models of predictive coding and active inference (Parr and Friston, 2013).

Box 4 **Mirror neurons: from action observation to emotion observation.**

The proposed emotional echopraxia achievable by an emotionally intelligent artifact can be interpreted as an extension of the brain's mirror neuron system—from action observation to emotion observation.

It has been established that when the brain observes another's motor behavior, this observation activates the same neural systems in the observer's brain as if she was herself performing the other's actions. This “reflected” neural structure is described in terms of mirror neurons, which are the neurons that fire in synchrony with the observer during action observation.

From the point of view of active inference, the role of mirror neuron system is to enable the brain to repurpose its generative model of motor behavior from understanding the self to inferring the intentions of another person (Kilner et al., 2007). In other words, because an agent knows their own intentions for moving, the mirror neuron system in action observation can repurpose this knowledge to interpret another's intentions for moving. This inference—that rests upon the mirror neuron system—is analogous to what we are proposing for emotion; namely, a mirror neuron system for emotion observation as oppose to motion observation.

### A Cultural Markov Blanket

Not only do biological agents conform to the free energy principle but ensembles of biological agents—brought together in the same environment (a niche)—should obey the free energy principle, too (Friston et al., 2015a; Constant et al., 2018). A model of free energy optimization provides a useful insight into how priors may be formed through evolution and culture, furnishing a new perspective on the origin of different emotional priors across people (Box 3). This process of optimization in evolution allows for an analysis of the way emotions are predicted and evinced differently across cultures, and an understanding of these processes is fundamental in an effort to build an emotionally intelligent agent (Heyes, 2018).

Ecologically, we speak of cultural niches within which organisms operate, and within these niches there are “affordances” referring to what the environment offers the organism: a shoe “affords” protection for his/her feet and “affords” a dog a chew toy (Ramstead et al., 2018). These affordances are examples of prior beliefs that have been encoded culturally, rather than arising independently in an organism. Crucially, the interaction between environmental affordances and human sensory states can be modeled with a Markov blanket (Ramstead et al., 2018). According to the free energy principle, over time and on average, human behavior will tend to reflect the statistical structure of the environment; i.e., actions will be guided by “encultured” beliefs (Constant et al., 2018). In fact, the structure of the human brain reflects the structure of its environment, i.e. environmental causes that are statistically independent are encoded in functionally and anatomically separate neuronal regions (Ramstead et al., 2018). The classic example of this is the separation of visual processing into a “what” and a “where” stream (Ungerleider and Haxby, 1994). From this perspective, the free energy principle can be applied to the internal states of the brain, the individual, or even the culture, where the levels of the nested internal states interact with the environment in different ways and to varying degrees (Kirchhoff et al., 2018).

There is a distinctive difference between the optimization performed by an adaptive agent's brain in real time, and that which occurs over generations. In ethology and evolutionary biology, the desired non-equilibrium steady state of phenotypes is defined through co-evolution and natural selection, meaning that it is a necessary consequence of natural selection, rather than the agent having to actively optimize policies to attain a specific non-equilibrium steady state (Frank, 2012; Campbell, 2016; Constant et al., 2018). This is plausible evolutionarily, because the model optimizes perceptual inference by allowing the organism to accumulate evidence across timescales and derive the best explanation for sensory data to achieve distal goals. Given the hierarchical time-scales inherent in the natural world, there is good reason to assume that organisms with this kind of cortical hierarchy would be favored by natural selection (Ramstead et al., 2018). Another way of phrasing this is that natural selection *is* Bayesian model selection, and that the best models (conspecifics) are those with the greatest evidence (lowest free energy) in a generation (Campbell, 2016).

If every organism is equipped with naturally selected Bayesian priors that have emerged from ecological niches and influence the species morphology, cognition and behavior, then an ecological niche will itself minimize free energy by “enculturing” its members so that they share common prior beliefs (Ramstead et al., 2018). Prior beliefs are therefore conditional on the unique ecological niches in which animals adapt through natural selection, development and learning, which explains why the inhabitants of different niches behave differently. At the same time, expectations within the same family, or species, are inherited and conserved across generations, and from this interplay emerges the phenotypical variety of life (Limanowski et al., 2015; Ramstead et al., 2018).

This resulting variety of behaviors due to evolution and adaptation in different ecological niches can offer insights into how humans express and interpret emotions. The way in which humans express emotions varies across cultures, differing in intensity for physical gestures, vocal emphases, or patterns of spoken and body language. This expression mechanism is an encultured prior, formed through cycles of updating prior beliefs to match expectations in each nested conspecific's generative model. Therefore, in order to successfully build an emotional inference agent, careful contextualization must be considered. The same sensory data (word choice, pitch, speed of articulation) could imply different kinds of emotional state for different people or in distinct social contexts. It is only by having a deep hierarchical model that accounts for this context that we might hope to draw sensible inferences about a person's emotional state from their voice. Part of this abductive capacity is being able to use context to work out which data features to “attend” to. If successful, a nested, recurrent model of emotion recognition and expression could be simulated using active inference, providing artificial intelligence with a way to accurately decipher emotions across different modes of expression.

### Sensory Attenuation

In active inference, the precision of prior expectations is crucial, because it regulates how confident the agent will be in its inferences and subsequent planning; i.e., behavior (Clark, 2013b). Increasing precision creates confident behavior and motivates action, whereas decreasing precision subverts action through uncertainty (Friston et al., 2012; Clark, 2013a; Hohwy, 2013; Seth and Friston, 2016; Badcock et al., 2017; Parr and Friston, 2017; Peters et al., 2017; Clark et al., 2018; Palmer et al., 2019). This must be balanced with the precision ascribed to predictions about the sensory consequences of behavior. Crucially, the active inference perspective on movement is that motor commands are simply predictions about the sensory consequences of a planned act. Action then corrects any discrepancy between predictions and sensory data. This raises an interesting problem—how do we start predicting movement related signals when we are not actually moving? In other words, if sensory data is consistent with being stationary, why do we update the world through action as opposed to updating our predictions? The answer to this is sensory attenuation.

Sensory attenuation occurs when brain adjusts the precision of its sensory input in order to properly modulate future action. In other words, the brain can act to suppress ascending prediction errors, thereby momentarily increasing the divergence between internal and external states in order to act (Blakemore et al., 1999; Shergill et al., 2005; Brown et al., 2013; Parees et al., 2014; Oestreich et al., 2015; Wiese, 2017). For example, one needs to be able to suppress the prior expectation of the position of their arm in order to be able to move their arm. In terms of psychophysics, sensory attenuation is a reduction in the intensity of sensory experience when action is self-generated, which can be measured with newer physiological methods by the amplitude of sensory evoked potentials (Brown et al., 2013).

The concept of sensory attenuation will become relevant in the following section, when we discuss a third wave of emotion recognition artifacts, which will potentially be able to engage with their users. In order to perform reciprocal prosocial interactions, the artifact will need to employ sensory attenuation in order to convey emotional information, and the user will do the same in order to infer the emotional state of the device. Creating an artifact that can accomplish this “turn-taking” behavior by performing sensory attenuation is significant in psychological research on disorders such as schizophrenia, because it is exactly a failure in sensory attenuation that is said to cause the associated delusions (Shergill et al., 2005; Brown et al., 2013; Parees et al., 2014; Quattrocki and Friston, 2014; Oestreich et al., 2015; Beedie et al., 2011). Its key role in communication and turn taking is exemplified in simulations of active inference in Kirchhoff et al. (2018).

Paranoid delusions, often involving the feeling of being threatened, arise in the brain's inability to perform sensory attenuation in the same way as non-schizophrenic brains. In order to overcome this, the brain increases its confidence (precision) in *high level beliefs* rather than in low level expectations. Thus, the brain becomes resistant to sensory evidence that contradicts its beliefs (Adams et al., 2013). When prior beliefs dominate perceptual inference, hallucinations such as hearing voices can occur. The brain can only understand this situation by falsely inferring, with a high degree of certainty, that an internal sensation is being generated by an outside agency, and this is a paranoid delusion (Friston et al., 2016b). For recent work on simulations of auditory hallucinations resulting from these false inferences, see (Benrimoh et al., 2018, 2019; Parr et al., 2018).

This anomaly in schizophrenic brains, possibly fueled by chemical imbalances in the neuromodulatory interactions between pyramidal cells and fast spiking inhibitory interneurons (Spencer et al., 2003; Sohal et al., 2009; Jardri and Deneve, 2013; Ranlund et al., 2016), sheds light on how schizophrenic patients misinterpret sensory information. Similarly, psychological phenomena of sensory misinterpretation arising in paranoia, depression, and autism can be explained with similar models (Lawson et al., 2017; Palmer et al., 2015; Clark et al., 2018). If one can simulate the way humans neurologically interpret emotions with generative models performing active inference, one could then adjust the simulation to characterize a schizophrenic, paranoid or autistic brain, and therefore better understand what the patient is inferring emotionally from their surroundings, gaining insight on how to approach patients with perceptual disorders in a formal way (Oestreich et al., 2015).

### Inferring Human Emotion

The remainder of this paper will be dedicated to comparing different machine learning approaches to emotion recognition and proposing second and third waves of emotional inference devices. Building a machine that can recognize and infer human emotions from facial expressions, body language and speech represents may be accomplished by replicating the brain's deep (i.e., hierarchical) inference—to recapitulate artificially what humans do instinctively (Vogt et al., 2008). This new form of artificial intelligence has clear applications in marketing, and also offers the potential for revolutionizing traditional clinical psychiatric practice; with the opportunity to model, understand, and possibly treat psychological disorders. Improvements in emotional inference devices could lead to studies of action observation that could have profound implications in disorders such as autism, potentially resulting in the creation of emotional companions that can update social priors (Lawson et al., 2017; Seth and Friston, 2016).

Any computational implementation of inference requires that the agent optimize a probabilistic model of how its “sensations” are caused. This requires a prior density that encodes beliefs about emotional states, a likelihood that specifies the ensuing sensory evidence, as well as the ability to act in order to modulate or confirm these expectations. The resulting updated predictions would then guide an agent to actively sample its sensory data to reach a non-equilibrium, low entropy steady state density. This entails matching a generative model as closely as possible to the environment (Friston et al., 2009). The resulting (approximate) isomorphism between the agent's internal model and external contingencies meets the necessary requirements of Conant and Ashby's Good Regulator Theorem; implying that the agent can be maximally successful at regulating environmental and sensory inputs (Conant and Ashby, 1970, 1991). With regards to inferring human emotions, the unknown causes (a.k.a., external states, hidden states, or latent variables) would be the emotional states; happy, sad, angry, disgusted, etc. (Panksepp et al., 1984; Craig, 2002; Barrett, 2006; Solms and Panksepp, 2012). The sensory states will be the medium through which the agent must infer emotional causes (speech, facial expressions, body gestures).

This categorization of emotional states raises an interesting question. Under the emotional inference perspective, emotions are hypotheses about the causes of data; but how do we define this hypothesis space? Broadly, there are two routes toward doing so. The first is to appeal to existing emotional taxonomies and to use this prior information to define alternative hypothetical emotions. The second is to appeal to structure learning, and to explore potentially very large hypothesis spaces to assess which of these hypotheses best account for the data at hand. Conceptually, this is like in clustering techniques, which posit a number of clusters and then find the parameters of each cluster that account for the data. The number of clusters in then optimized by penalizing excessively complex solutions (with many clusters). Ultimately, we anticipate using some combination of the two, using prior information to constrain the space of plausible emotions and optimizing these through structure learning.

Once the emotional states have been classified an agent must engage a combination of explorative and exploitative behavior in order to perform an inference about the current state. The present standard of emotional inference rests on a supervised learning approach, yet we suggest that the coming stages of emotional devices will use an active inference approach, which can eventually be improved upon to synchronize generative models and result in artifacts capable of emotional engagement. These distinct approaches speak to three waves of emotion recognition devices, from the current state of the art to emotional artifacts in the decades to come.

### Wave One

The current state of the art—in terms of emotional inference devices—consists of deep learning models, often implemented in phone apps using cameras (Alshamsi et al., 2016). To train these agents, an enormous amount of visual information is collected, and the agent is trained on which emotions the photographs display. Over time, the agent begins to recognize patterns within this visual information—and attempts to infer emotions accordingly (Alshamsi et al., 2016).

The most advanced form of deep learning uses a variational autoencoder that has a particular deep structure. The supervised agent is initially placed in a controlled environment to facilitate learning, and then placed in an uncontrolled environment to assess performance (Jeon and Shin, 2019). The sophistication of this supervised neural network derives from its implicit generative model which underlies an explicit representation of uncertainty, allowing for the backpropagation of errors (Jeon and Shin, 2019). Interestingly, the objective function for learning how to recognize or encode particular emotions is exactly the same as the variational free energy that underwrites active inference (Winn and Bishop, 2005).

During learning, the agent does not perform any actions but rather is passively pushed toward the “correct” classification in the presence of exemplar (i.e., training) datasets. This leads to learning a distribution in which the sensory input as well as the resulting “correct” response are more probable in conjunction with one another. The agent learns to understand the causal structure of the training environment, inferring the causes of sensory states, and these inferred causes induce prior expectations, which the agent will then retain for its test phase in the uncontrolled environment (Jeon and Shin, 2019). When the parameters of the agent's internal model have converged to the parameters of the controlled environment, the agent is successfully programmed to expect to give the correct classification of the desired emotional state based on the environmental signal (Gan et al., 2017). The result is an agent that has an optimum policy for each situation that it is confronted with.

So far, the above approach has not been successful in inferring emotion as efficiently as a human being. This is because of three main reasons; firstly, from a structural point of view, the supervised backpropagation of errors in a neural network only supports an implicit generative model, and thus the agent lacks explicit control of prior beliefs that are important in generating predictions of emotion. Second, in supervised learning, the objective function (the function that the algorithm aims to optimize) is a scalar function of an outcome, usually a classification of accuracy, rather than a functional of beliefs (Gan et al., 2017). This means that supervised learning models do not have an explicit or proper way to accommodate uncertainty, nor can have beliefs about the consequences of action. Thirdly, deep learning models never perform action to optimize belief updating all learning, and they are unable to actively elicit responses from their users.

The eliciting of emotional responses is the most essential component that deep learning lacks when it comes to emotional inference. If one cannot act, one cannot learn how the user will react to an action, which is most likely explains why human beings are so efficient at gauging emotional states; consider how much a slight eye roll or twitch of the head can communicate from one individual to another (Parr and Friston, 2019). We argue that an active agent—operating under an explicit generative model—with an objective (variational free energy) functional of beliefs will allow for machine-human interaction, setting the stage for the second wave of emotional recognition technology, in which an agent will be able to perform active inference to evoke emotional responses from its user.

### Wave Two

Active agents are compelled to sample outcomes that are relevant to the task at hand, even if they are associated with high uncertainty, which is an emergent behavior under the minimization of expected free energy (Barto et al., 2013; Schwartenbeck et al., 2013, 2019; Friston et al., 2015b, 2017c; Limanowski et al., 2015). An agent will first select between plausible policies, and then choose those that resolve the most uncertainty about the parameters of its generative model; here, between its emotional priors and latent emotional states. Following this, the agent will be placed in an uncontrolled environment where it would infer emotional states from external signals based on its prior expectations and the emotional affordances of particular behaviors (Smith et al., 2019a).

Crucially, the active inference formulation of the problem frames it as an optimization problem. The quantity being optimized is Bayesian model evidence (which is maximized) or its complement, surprisal (which is minimized). This gives us a simple way to evaluate performance. The greater the model evidence, the better the categorization. To unpack this further, model evidence may be decomposed into two parts: accuracy and complexity. As the accuracy with which the model predicts sensory data increases, so does the evidence. However, this is penalized by the complexity. As complexity increases, the model evidence declines (unless there is a compensatory increase in accuracy). This tells us that the best emotional categorization will be the simplest that accurately accounts for the data available to our system. For an example of this approach in the context of inferring abstract rules, see (Friston et al., 2017a).

The first advantage of using active inference is that the agent can leverage the potential epistemic richness of very different sorts of data, beyond just profiles of faces. An active inference agent will have an explicit generative model of emotional states, and therefore it will generate predictions in multiple modalities present at various temporal scales, such as facial motion, heart rate, hyperemia in the face, posture, etc. (Seth and Friston, 2016).

This highlights the important distinction between the implicit generative model of a deep architecture explained above, and the explicit generative model of active inference (Buckley et al., 2017). A deep architecture will first gather all of the available data and then attempt to identify a small number of latent nodes at the top of the [de]convolutional network that represents emotional states (happy, sad, angry, etc.). Conversely, the generative model of active inference is the inverse of this deep architecture. In other words, starting from these latent emotional states, the model generates all of the high dimensional consequences, including the modalities referred to above, which will be contextualized by being in the current emotional state (Friston, 2009). Therefore, everything it can predict from an emotional state becomes a potentially useful data feature—that will inform and update the model.

The nature of this inverse model means the agent will learn to understand infer “where to look” next in order to resolve the uncertainty about the emotional state of the subject. This epistemic foraging, or “active vision,” implies that the agent will learn to deploy its attention only on emotionally salient parts of the face (Smith et al., 2019b). Therefore, rather than wasting computer floating point operations on the entire visual field, the agent can imitate the way a human will saccade to garner visual information, focusing only on the eyes, mouth, nose, and possibly forehead. This will result in a simulated agent that can correctly deduce emotional states that it has not yet encountered, by using its internal model to choose the most likely (and therefore correct) outcome. This model has the advantage that a smaller dataset with shorter training time may suffice, as data are autonomously selected to optimize the model (Friston, 2009).

This generative model can lead to all sorts of learnable features that can potentially provide insight into psychiatric disorders. For example, if a psychiatrist is unsure what depression looks like, they can train the generative model by providing it with the emotional states of a depressive inventory, and it will learn to associate this profile of depressive-like scores with a cause (Cullen et al., 2018). Instead of initially presenting labels of depressed or not depressed, which is what supervised learning would do, the generative model is able to learn the nature of its environment on its own, and then come up with a specific set of complex responses that correspond to one or more emotional states.

In addition to this, an artifact from the second wave will also engage in active *learning*, allowing it to then ‘learn on the job’ from the user's reactions to its perturbations (Friston et al., 2016a). This might involve equipping it with the opportunity to take actions to elicit responses from the person it interacts with (e.g., asking questions, or for feedback). By selecting courses of action that minimize expected free energy, these actions would be used to resolve uncertainty about the model over time, and about emotional states at any given time. In addition to the principled underpinnings of active inference, it is this capacity to autonomously optimize beliefs about the world through selecting the best (epistemically valuable) actions that renders this approach distinct from alternatives (Friston et al., 2016a).

Eliciting emotional responses from the user can be accomplished most efficiently with the conveyance of visual (and perhaps auditory) information, as opposed to other sensory signals or collecting personal data. This immediately suggests that the second wave of emotionally intelligent artifacts will require a visual representation of a face with which the artifact can perform actions (eye movements, smiling, blushing etc.) and observe how the user responds. This is crucial, because—if the inference device is uncertain about which emotional state the user is in—free energy minimization will enable it to display the expression (epistemic perturbation) that will resolve the most of this uncertainty, given the lexica of emotional states that the device is equipped with (Smith et al., 2019a). In other words, the device will continuously select from its available actions to maintain a precise (i.e., confident) inference about the emotional state of the user, very much like a human would do (Friston et al., 2016a).

Due to the ability of the device to gauge the emotional states of the user in a matter of seconds and learn from the user's responses, the artifacts of the second wave will prove to be significantly superior to those of the first wave. These devices have clear applications in marketing, such as understanding how people interact with certain products. Perhaps more importantly, they are relevant in terms of e-health, potentially allowing for automated diagnosis of psychological or emotional disorders.

### Wave Three

A potential improvement upon the above active inference model is an agent that actively engages with the user. In wave two, the device infers the user's mood by performing active learning and inference under a generative model of the user's emotional states. This is conceptually different from an artifact that the user can actually engage in emotional exchanges; meaning that the user's generative model will also begin to predict the artefact's behavior. This machine-to-human reciprocal emotional interaction may eventually be feasible, as suggested by recent research in songbird simulations that demonstrate how communicating agents that have the same underlying brain structure can synchronize their generative models (Friston and Frith, 2015a). This conceptual leap defines the difference between an *emotionally intelligent* artifact in wave two, and an *emotional* artifact in wave three.

Recent work proposes an active inference approach to communication, explaining how in order to communicate, two agents must be modeling each other in an infinite regress (modeling you modeling me modeling you and so on), and through this coupling the agents adopt the same generative model, i.e., they adopt generalized synchrony between internal brain states (Friston and Frith, 2015a). This work demonstrates how through sensory information, an agent can gain insight into the internal model of another.

Furthermore, this model of communication is intricately linked with the sensory attenuation discussed in section Emotional inference, selfhood, and the human brain, demonstrated through a simulation of a songbird that must attenuate the sensory consequences of acting in order to act (in order to sing). In a communication context, this underscores the fact that one cannot listen and speak at the same time; further supported by a simulation of two songbirds who sing to each other, both undergoing this intermittent sensory attenuation in which they listen to each other. By synchronizing their internal models, generalized synchrony emerges and communication results (Friston and Frith, 2015a). In the context of emotion recognition, this demonstrates the possibility of synchronizing internal brain states of the emotional device and its user, such that the device and user are both attending to and attenuating sensory information in order to properly cause and predict another's emotional states (Friston and Frith, 2015a).

This form of an artificial emotional companion can only be achieved once the circle is closed between human and machine; meaning that not only does the machine actively infer emotions from the human, but the human actively infers emotions from the machine as well. Therefore, it would be necessary for the artifact to process interoceptive signals, and possibly verbal signals as well. The artifact would need to learn this implicit “emotional lexicon” through a human agent; i.e., it would start off with an emotional dyslexia and then learn from interactions with its user. The hypothesis is that through this training, the artifact would eventually converge to the user's generative model of emotional states. The potential convergence of a “student's” generative model upon the generative model of a confident “teacher” (of the same type) is exactly what the bird song simulations above aimed to demonstrate (Frith and Wentzer, 2013; Friston and Frith, 2015b).

**Figure 3:**
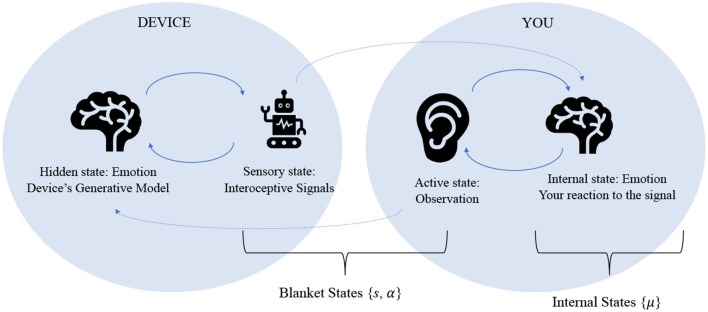
This figure represents the essence of a third wave emotional device, in which the artifact and user are able to synchronize their internal models such that they become each other's external states. In this sense, the device itself is emotional, and the human interacts with it emotionally—in the same manner as it would with another human.

This convergence of generative models can also be interpreted in terms of coupled Markov blankets, demonstrated in Figure 3. The Markov blanket description in section Theoretical overview represents a single agent's separation between “self” and “other,” in which the conditional independence of the blanket states separates the agent's internal world from its external world through active and sensory states. A representation of synchronized generalized models, on the other hand, would exclude external states entirely, because the “other” now becomes another agent's internal states (Friston and Frith, 2015a).

In wave three, the epistemic foraging for disclosing information about the user's emotional state requires the additional constraint that the artifact shares the same generative model that it believes the user has. Under these synchronized generative models, the artifact will engage in active inference to resolve uncertainty and effectively be able to interact emotionally with the user (Seth and Friston, 2016). This prosocial and reciprocal interaction can be interpreted as the machine possessing an emotional echopraxia, i.e., it will effectively mirror its user's emotions (Ainley et al., 2012).

Finally, the emotion recognition architecture of the second wave is largely epistemic, so it is not necessary to implement explicit goals or preferred outcomes in the generative model. Without preferred outcomes, the device learns a set of minimally complex emotional states, under the prior that the user's generative model is congruent to that of the device. However, once the device and human have established a generalized synchrony, it would be possible to implement prior preferences in the agent's generative model—so that it can steer the emotional synchrony to a specific desired emotional state (happy, calm, etc.). This holds promise in therapeutic applications, because the model will be able to guide humans to interact in certain ways by inducing a desired state in the brain as the best explanation of the sensorium (Seth and Friston, 2016). This has potential clinical applications in disorders such as autism and schizophrenia, in which patients experience a failure of interpersonal inference, false inference about the intentions of others, or failure to develop proper theory of mind (Lawson et al., 2017). By training synchronized generative models to prefer desired states, the user could potentially learn how to overcome neurological tendencies via the device.

## Discussion

Several accounts of free-energy minimizing models of communication and personal emotional awareness serve as a foundation for further and more complex representations of how emotions can be taught and learned socially, in two person (dyadic) interactions or group situations, as well as how one can (or cannot) develop an emotional model based on sensory information throughout childhood and adulthood (Smith et al., 2019b). These models demonstrate certain important intricacies that must be carefully considered when building such an emotional inference device, such as the precision of priors, contextual information, and Bayesian filtering.

A good model of an emotionally aware agent depends on precise priors over external factors, as well as the ability to quickly switch between contextual information. In order to model a complex form of emotional communication between individuals or in society, the priors become supremely important, due to their direct influence on the confidence with which the agent can act on its beliefs. In other words, not only do the priors need to be accurate, they also need to be precise in order for the agent to have sufficient confidence to act accordingly.

Furthermore, while there may be some universal micro-expressions or vocal patterns that the agent can pick up on—as priors that are context-independent—most human communication and expression is heavily dependent on situational factors. This intricacy arises from the complexity of the cortical hierarchy and the continuous exchange between environmental and internal states via sensation and action, making each exchange somewhat unique. For this reason, to create a successful AI that can decipher emotion, the environmental priors must include contextual states that influence how other environmental states will generate respective data, so that the generative model will treat sensory signals uniquely based on the situation in which they are presented.

Research in songbird simulations demonstrates how generalized Bayesian filtering allows the brain to maintain distinct generative models for different contexts; i.e., to interact with—and make inferences—about several different agents about who they are and the content they are communicating (Isomura et al., 2018). Being able to continuously fluctuate between different internal models based on changing sensory information allows an agent to harvest the most relevant information by continuously switching to the best explanation for a given sensation. This further suggests that an active inference approach to communication and inference can account for the complexities of animal interaction; not only by synchronizing internal models between communicating agents, but also through the ability to possess multiple models simultaneously for group interactions. In turn, this suggests a basis for how cultural niches update priors and affect behavior and expectation (Heyes, 2018; Veissiere et al., 2019).

Finally, when it comes to emotion recognition, this type of inference is also performed by individuals that have their own emotional awareness. This could draw upon recent advances in the formal modeling of emotional inference using active inference and Markov decision processes (Smith et al., 2019b). This research also covers emotion conceptualization in childhood (Ainley et al., 2012; Fotopoulou and Tsakiris, 2017); i.e., how one can form a model of emotion recognition without any initial prior beliefs or expectations about emotional content. This line of work suggests that as long as an agent is provided with a fairly consistent sample of emotional experience in “childhood”—that allows it to form relatively precise priors over time—it can reach a 100% accurate model of emotion recognition (Smith et al., 2019b). Moreover, the model continues into “adulthood” and explains how new emotions can be learned and how this affects existing emotional priors, while demonstrating the extent to which unstable emotional environments of childhood can bias or inhibit emotion conceptualization (Smith et al., 2019b). This work stands as a proof of concept for an active inference approach to emotion recognition and the wider scope of what it could explain about the emotional human brain throughout development.

The potential applications of an emotionally intelligent system are diverse. Opportunities in psychological research have already been mentioned, in terms of adjusting a computer model to simulate a brain with a psychological disorder such as schizophrenia or autism—aiming to understand how patients interact with emotive content in their internal models of the world (Beedie et al., 2011). Furthermore, it could be used for anthropological and psychological research in understanding how different cultures express emotions differently, which could be useful for economic and political matters; e.g., engaging with other markets or cultures in an informed way.

In addition, effective emotion recognition technology is already being looked into for industry and government applications; such as in classrooms—to understand how different students learn differently—in order to improve educational methods (Toor, 2017), as well as retailers such as Walmart that hope to implement emotion recognition technology in stores to understand how humans react to certain products in the aisles (O'Shea, 2017). The current applications implement the first wave of emotion recognition devices; namely, deep learning technology. However, if research is pursued in the direction of active inference, this may establish the future of accurate emotional inference across cultures, personalities and stages of development.

## Conclusion

It is important to return to the nature of the free energy principle as a scientific construct. The free energy principle was formulated to explain how adaptive agents learn, and to encompass all of self-organizing entities into a unifying scientific theory. So, where does this leave us, as humans trying to understand ourselves from the inside out?

Humans are self-organizing creatures with an abundance of sensory information that we must carefully filter in order to properly operate in the world around us. Because of each of our unique paths through evolution, genetics and the environment, we process sensory data and behave in different ways, and for those of us with mental disorders, these distinctions are much more severe. Nevertheless, the Bayesian brain and free energy minimization provide us with a scientific approach for all forms of perception, inference and behavior—in the same formal framework. This is a new and deeper answer to the question “Why?” when it comes to human behavior, psychology, and society, and if implemented correctly, could potentially be the starting point for a new chapter of human knowledge, with a deeper insight into ourselves, as well as more effective, intelligent and “humanoid” computers.

For this reason, it seems that an interesting way to perform future research on derivations of the free energy principle in psychology and machine learning could be to integrate the Bayesian brain model for perception into emotion recognition. Through building complex generative models—in which simulated agents can actively infer emotions—in the same way that a human does throughout its lifetime, we could speed up emotional learning artificially and better understand its influences and patterns. In addition, if we eventually manage to synchronize an artificial generative model with that of a human brain, we could potentially establish machine-human emotional connections that could commence a new chapter in psychiatric diagnosis and treatment. In sum, the information that these potential simulations can provide will allow the members of our species to answer long-sought questions about emotional development, social interactions, and disorders of the mind.

## Author Contributions

DD wrote every draft of the paper and made all of the figures. TP edited the paper numerous times for suggestions, and he rewrote quite a bit of the theoretical overview (Theoretical overview). KF contributed mainly to the content of Inferring Human Emotion through explanation in person, and he also went over the paper for final edits and corrections.

## Conflict of Interest

The authors declare that the research was conducted in the absence of any commercial or financial relationships that could be construed as a potential conflict of interest.

## Footnotes

^1^Listening is used here in the sense of orienting to a sound. Active states of this sort could be framed in terms of motoric (turning one's head) and mental (attentional) states. See Metzinger, 2017 and Friston, 2018.

## Glossary:

- Generative model: or forward model is a probabilistic mapping from causes to observed consequences (data). It is usually specified in terms of the likelihood of getting some data given their causes (parameters of a model) and priors on the parameters.
- Markov Blanket: a statistical boundary that renders everything outside the blanket conditionally independent of everything inside of it.
- Self-organization: the process by which the local interactions between systemic states spontaneously develop order.
- Bayesian Brain: Bayesian approaches to brain function investigate the capacity of the nervous system to operate in situations of uncertainty in a fashion that is close to the optimal prescribed by Bayesian statistics.
- Entropy: The average surprise of outcomes sampled from a probability distribution or density. A density with low entropy means that, on average, the outcome is relatively predictable. Entropy is therefore a measure of uncertainty.
- Surprisal: The negative log-probability of an outcome. An improbable outcome (for example, water flowing uphill) is therefore surprising.
- Prior: The probability distribution or density of the causes of data that encodes beliefs about those causes before observing the data.
